# Reconstruction of Simple Incomplete Syndactyly of the Foot

**Published:** 2018-12-03

**Authors:** Anthony M. Kordahi, Laurel S. Karian, Paul J. Theratil, Mark S. Granick

**Affiliations:** Rutgers–New Jersey Medical School, Newark

**Keywords:** syndactyly, foot, Z-plasty, congenital, web space

**Figure:**
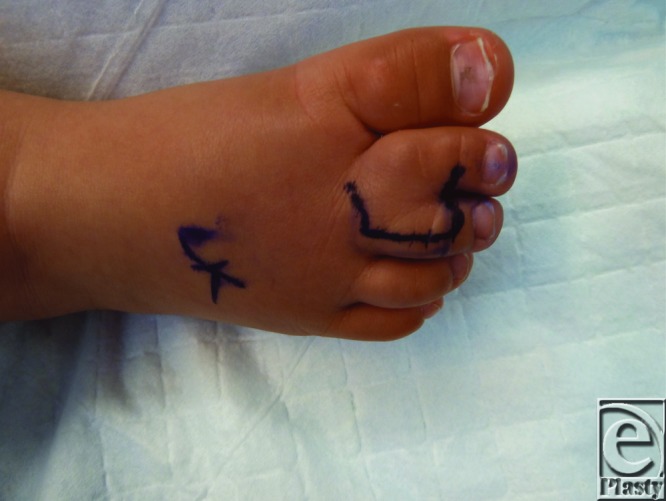


**Figure:**
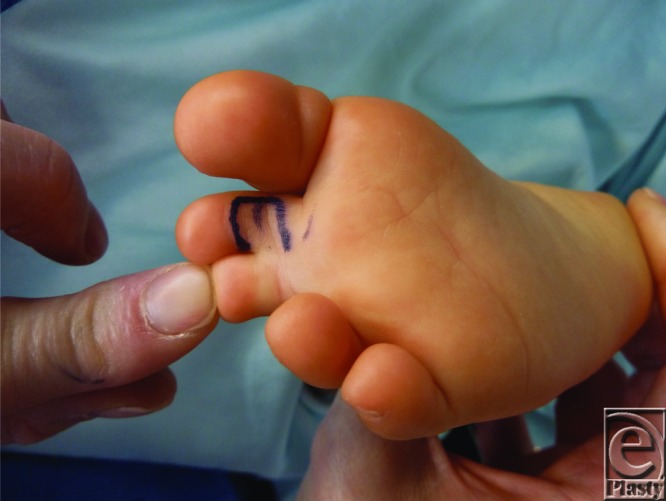


**Figure:**
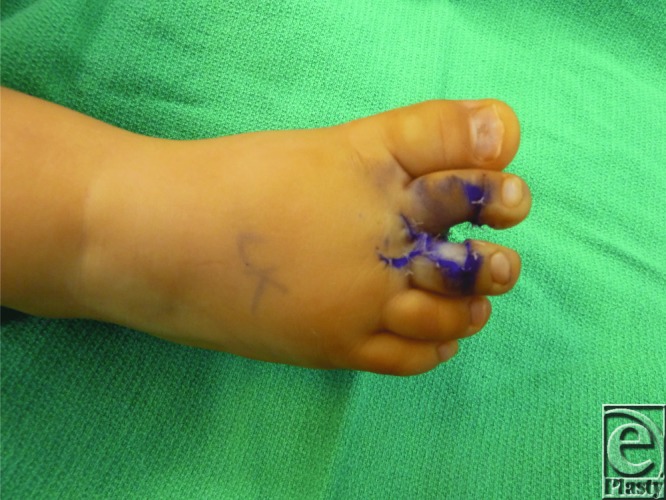


**Figure:**
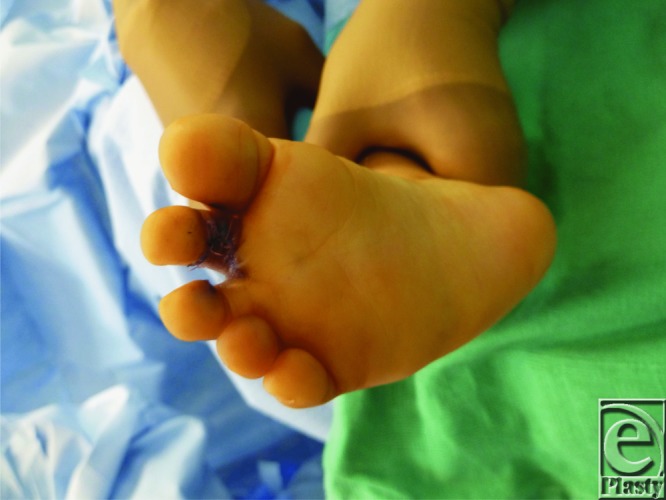


## DESCRIPTION

A 10-month-old male child presented to the plastic surgery service for simple incomplete syndactyly repair of the second and third toes on his right foot.

## QUESTIONS

1. What is the epidemiology associated with syndactyly?
2. Is syndactyly associated with any congenital syndromes?
3. What are the treatment options available for syndactyly and some common complications?
4. How does the treatment of syndactyly of the foot differ from that of the hand?

## DISCUSSION

1. Syndactyly is a congenital anomaly that is caused by a failure of apoptosis during embryologic development, leading to the persistence of web space tissue. It can be classified as either simple, when there is only soft tissue involved, or complex, when there is bone involvement. Complete or incomplete refers to whether or not the entirety of the digits is fused. Its incidence is approximately 1 in every 2000 live births, being twice as common in the male population, and 10 times more common in whites than in blacks. In the foot, the most frequently afflicted web space is that of the second and third toes. While the majority of syndactyly cases are isolated findings, up to 40% of cases have some form of genetic inheritance. Most commonly, syndactyly appears to be of autosomal dominant inheritance with incomplete penetrance when there are no other congenital anomalies present.^1^^,^^2^
2. Syndactyly can be associated with a variety of congenital syndromes. These syndromes can be divided into acrocephalosyndactylies, which include Apert and Crouzon syndromes, and acrocephalopolysyndactylies, including Carpenter syndrome. Other common causes of syndactyly include Poland and amniotic band syndromes.^3^^,^^4^
3. Treatment of syndactyly varies on the basis of patient presentation. The basic principles of syndactyly repair include the separation of the digits, skin coverage, and the return of functionality to the fingers.^5^ For more complex cases where there is bone involvement, early treatment involving division of the bone is warranted to prevent joint deformities. When there is no bone involvement, the use of full-thickness skin grafts, most commonly from the groin, is one option that has gone out of favor due to inferior aesthetic results and increased technical difficulty of the procedure, especially in the young patient population afflicted by this condition. The use of local flaps, such as Z-plasties, to provide for adequate coverage is appropriate because of their increased ability to prevent scar contracture. Another option comprises 2 flaps involving the dorsal and plantar surfaces of the distal metatarsal.^6^ Common complications of syndactyly repair include damage to the neurovascular bundles that run along the ulnar and radial aspects of the digits, which can result in vascular and sensory deficits to the distal aspect of the digits that can lead to flap necrosis, infection, and functional deficits due to decreased sensation. Another complication includes web space creeping due to scar contracture, resulting in the reformation of a web space affecting function.^7^
4. The management of syndactyly of the hand is crucial because of the intricate utility of the hands in everyday life. Restoration of function is of utmost importance, with aesthetic concerns being secondary considerations.^5^ Adequate surgical planning to avoid scar contracture is necessary due to its potential to cause functional deficits. When contemplating the treatment of syndactyly of the foot, the primary functions of the foot are not affected by retained webbing between the toes. The goal of attaining a functional foot involves being able to provide adequate surface area for ambulation and balance. Thus, aesthetic interests become the primary treatment goal of foot syndactyly repair. Our patient's procedure involved 2 rectangular transposition flaps, one on the dorsal surface and one on the plantar surface of the web space, to repair the second and third toes, providing an aesthetically pleasing result.
